# The Role of Trauma in Early Onset Borderline Personality Disorder: A Biopsychosocial Perspective

**DOI:** 10.3389/fpsyt.2021.721361

**Published:** 2021-09-23

**Authors:** Paola Bozzatello, Paola Rocca, Lorenzo Baldassarri, Marco Bosia, Silvio Bellino

**Affiliations:** Department of Neuroscience, School of Medicine, University of Turin, Turin, Italy

**Keywords:** abuse, borderline personality disorder, etiopathogenesis, early onset, comorbidity, trauma

## Abstract

The role of childhood trauma in the development of borderline personality disorder (BPD) in young age has long been studied. The most accurate theoretical models are multifactorial, taking into account a range of factors, including early trauma, to explain evolutionary pathways of BPD. We reviewed studies published on PubMed in the last 20 years to evaluate whether different types of childhood trauma, like sexual and physical abuse and neglect, increase the risk and shape the clinical picture of BPD. BPD as a sequela of childhood traumas often occurs with multiple comorbidities (e.g. mood, anxiety, obsessive-compulsive, eating, dissociative, addictive, psychotic, and somatoform disorders). In such cases it tends to have a prolonged course, to be severe, and treatment-refractory. In comparison with subjects who suffer from other personality disorders, patients with BPD experience childhood abuse more frequently. Adverse childhood experiences affect different biological systems (HPA axis, neurotransmission mechanisms, endogenous opioid systems, gray matter volume, white matter connectivity), with changes persisting into adulthood. A growing body of evidence is emerging about interaction between genes (e.g. FKBP5 polymorphisms and CRHR2 variants) and environment (physical and sexual abuse, emotional neglect).

## Introduction

Borderline personality disorder (BPD) is a severe and heterogeneous disorder characterized by chronic instability, with episodes of severe affective and impulsive dyscontrol, interpersonal and identity disturbances (1). Patients with BPD show a marked emotional sensitivity with the incapacity to modulate intense emotional responses and inadequate return to emotional baseline (2).

The prodromal signs and symptoms that prelude to later personality pathology are already present in very young age, in particular in adolescence (3), meaning that BPD does not abruptly emerge during adulthood.

Prevalence rate of BPD in non-psychiatric population is ranged between 0.7 and over 5% (4, 5) while in clinical settings reaches 10% of all psychiatric outpatients and 15–20% of inpatients (6). BPD is characterized by severe functional impairment, intense use of health services, medications, and a suicide rate of 10–50 times higher than the rate in the general population (7).

Psychodynamic theories suggested that BPD arises from precocious distortions in object relations (8) and characteristic patterns of attachment (9), inducing an intolerance of aloneness, hypersensitivity to environmental stimuli, expectation of detached and hostility from other, and loss of positive memories of dyadic relationship (10).

From early attachment experiences, subjects gain important information about their identity and abilities to regulate inner experiences and behavioral strategies for maintaining proximity to others. Aggression or neglect from caregivers may be experiences contributing to compromise a realistic and balanced view of self and others (10). So, an insecure model of attachment with caregivers is internalized and could be at the origin of the expectancies for future relationships characterized by abuse or rejection (11). BPD occurs in a precocious context of intolerance toward the manifestations of private emotions during childhood (12). So, children exposed to these unfavorable conditions are unable to recognize, regulate, and tolerate emotional responses and they fluctuate between extreme emotional lability and emotional inhibition. In 30% up to 90% of cases BPD is associated with abuse and neglect in childhood and these percentages are significantly higher than those registered in other personality disorders (13–15). Some authors proposed affect regulation difficulties as central mediator in the relationship between childhood trauma and BPD (16). Interpersonal dysfunctions are a result of early scarcity of emotional closeness or responsibility from caregivers in early childhood (17).

Similarly to other psychiatric disorders, the most acknowledged etiopathogenetic theory of BPD suggested that this disturbance was produced by the interaction of biological and psychosocial factors (17–19), in particular biologically based vulnerabilities (temperamental features, genetic polymorphisms) and adverse environment (traumas) during childhood or adolescence. These traumatic experiences could be also associated to neuromorphological abnormalities and to neuroendocrine changes (i.e. hypothalamic-pituitary axis (HPA) activation) that are observed in patients with early BPD and abuse/neglect history (20).

The aim of this review is to provide an updated overview on different types of traumatic events (sexual and physical abuse, neglect, and bullying) that combined with temperamental, genetic, and neurobiological factors may contribute to early manifestations of BPD.

We searched in Pubmed database studies focused on borderline symptoms and disorder in adolescents up to 20 years old, published between 2000 and 2021 and using the following terms: (borderline personality disorder) AND (child OR adolescent OR teen OR young) AND (trauma OR abuse OR maltreatment OR neglect OR bullying) AND (temperament) AND (environment) AND (epigenetic factors OR neuroimaging OR neurobiology) AND (suicide OR self-injury) AND (cPTSD). Eligibility status for articles was defined by the initial screening of trials on the basis of title and abstract. Papers that passed the initial screening were further selected on the basis of a careful examination of the full manuscript content. The review considered only articles written in English. We included the following type and number of studies: 30 longitudinal, 12 retrospective, 9 cross-sectional, 1 randomized controlled trials. Assessment instruments more commonly used in the trials were: the Borderline Personality Features Scale for Children (BPFS-C) and a recently developed parent report version of the same measure (BPFS-P); the Revised Diagnostic Interview for Borderlines (DIB-R); the Child Interview for DSM-IV Borderline Personality Disorder (CI- BPD); the Child Interview for DSM-IV Borderline Personality Disorder. Number of studies participants ranged between 12 and 7,771. Age of patients ranged between 9 and 21 years. Five studies included only females. Participants were mainly Caucasian.

## Factors Associated to Early Onset of BPD: Temperamental Traits and Environmental Context

### Temperamental Factors

Intrapsychic factors, including temperamental characteristics and personality traits in childhood and adolescence, must be investigated to recognize predictors of BPD at an early phase (3). There is a general consensus that temperamental vulnerabilities combined with childhood adversities play a role in the development of BPD traits (12, 21, 22). Researchers identified several temperamental traits in children or adolescents, including affective instability, negative affectivity, negative emotionality, inappropriate anger, poor emotional control, impulsivity, and aggression, that could predispose to borderline personality disorder (3, 23, 24). Model proposed by Cloninger for personality pathology underlines the role of temperamental characteristics in BPD development (25). Some authors found that high harm avoidance and novelty seeking (in combination with childhood traumatic experiences and adolescent psychopathology) can be considered predictive of early onset BPD (26). In a randomized controlled study that compared 33 BPD adolescents with 35 clinical controls and 31 healthy subjects, it was observed that high harm avoidance and novelty seeking, together with low reward dependence represented a vulnerability for BPD onset (27).

In particular, novelty seeking is the temperament dimension that was specifically linked with BPD and differentiated BPD patients from non-clinical patients, patients without personality disorders (PDs), and patients with other PDs (including diagnoses of cluster B disorders) (28).

Among other temperamental traits, aggression at a very juvenile age was associated with precocious onset of BPD (24, 29, 30). Vaillancourt et al. found in a prospective study performed in 484 children and adolescents that aggression was a predictor for the early diagnosis of BPD (at 14 years) with some gender differences. In particular, relational aggression was the main predictor in boys, while physical aggression was the strongest predictor in girls. The same authors suggested that negative emotionality, in terms of negative affectivity and poor emotional control, was another temperamental trait often associated to early BPD (31). Several studies designed to evaluate how negative emotionality and other traits, such as affiliation, constraint, and agency, might impact onset of BPD showed that negative emotionality and low constraint predicted onset of BPD at younger age (32, 33), as well as the association of negative affectivity with impulsivity and lower sociability in childhood (34, 35).

Two different longitudinal studies by Gratz and Tragesser identified that three tightly connected traits—low self-control, impulsivity, and affective instability—can be considered predictors of early onset BPD (34, 36). One study investigated the impact of the temperamental trait of anger on precocious BPD (37). Crawford and colleagues showed a significant association between anger trait and early BPD symptoms in a large sample of 766 children who were followed for 20 years (38).

Available data sustained the hypothesis that temperamental factors may increase vulnerability for the development of BPD, but they are not sufficient to predict this disorder and need to interact with environmental negative factors to induce it. Several investigations indicated that the interaction of temperamental vulnerabilities with environmental experiences [i.e., distressing childhood experiences within the context of the family (12, 22)] is a significant risk factor for the development of BPD in early age (21, 37).

### Environmental Factors

Environmental factors that have been identified as predisposing conditions for early BPD encompass familial maltreatments (abuse and neglect), psychopathology of family members, and parent-child conflicting relationship. It has been supposed that the development of cognition and affectivity, the integration of thinking and emotions, and the ability to discern emotional states are disturbed by early trauma with consequent post-traumatic reactions, dissociation, and alexithymia (39).

Children who suffer maltreatments may infer negative characteristics of themselves and others and deduce that they are intrinsically unacceptable and deserve maltreatment. This assumption may lead to see themselves as “helpless, unlovable, or weak and to view other people as dangerous, rejecting, or unavailable”. So, abused children may internalize negative perception about themselves, others, and about relationships with other people (40).

Childhood familial maltreatments may foster BPD through insecure attachment. Abused children tend to “blame themselves for their maltreatment when the perpetrator is an attachment figure” (41). In particular, more severe and chronic experiences of maltreatment may produce a negative image of oneself with consequent expectation to be abandoned (i.e., attachment anxiety) and a simultaneous negative perception of others as unworthy of being trusted (i.e., attachment avoidance) (40). Some studies showed that primarily attachment anxiety, rather than avoidance (more related to father's maltreatment), plays a role in the relation between child maltreatment and the development of precocious BPD symptoms.

Findings from studies focused on maternal psychopathology emphasize the importance of considering the diagnosis of BPD in mother as predictor of BPD onset in adolescence (around 15 years of age) (42). Maternal externalizing disorders, characterized by poor impulse-control, rule breaking, aggression, and impulsivity were significantly associated with early BPD in offspring (43). Maternal anxiety and depression during pregnancy predict early BPD in sons or daughters. (44).

Among familial relationships, role confusions and disoriented behaviors in parent-child relationship were found in patients with early BPD symptoms, in particular self-injuries in adolescence (45). Moreover, dysfunctional familial relationships characterized by induction of guilt, psychological control, and triangulation (children who have a role of mediator in the parental marital conflicts), were observed in large samples of adolescents with severe behavioral and emotional disorders who had already showed BPD symptoms in childhood (46).

Studies on temperamental and environmental factors associated to early onset BPD are presented in Table 1.

**Table 1:** Studies on temperamental and environmental factors associated to early onset BPD.

**Temperament and environment**	**Study design**	**Recruitment age (mean or range)/patients (*n*)**	**Outcomes**
Fossati et al. (28)	Retrospective study; Clinical outpatients and community population	44 BPD 207 CC 206 HC	Novelty seeking is the temperament dimension specifically linked with BPD
Joyce et al. (26)	Longitudinal study; Clinical outpatients	18–35 yrs 180 MDD	High novelty seeking and high harm avoidance in combination with childhood experiences and psychopathology predicted BPD
Crawford et al. (38)	Longitudinal study; Community population	At birth 766 children	Significant association between anger/tantrum traits and precocious BPD symptoms
Gratz et al. (36)	Longitudinal study; Community population	9–13 yrs 263 children	Low self-control, impulsivity, and affective instability can be considered predictors of early onset BPD
Tragesser et al. (34)	Longitudinal study; Community population	18–20 yrs 353 adults	Low self-control, impulsivity, and affective instability were predictors of early onset BPD
Belsky et al. (23)	Longitudinal study; Community population (ERLTS)	At birth 2,232 twins	Affective instability, negative affectivity, negative emotionality, inappropriate anger, poor emotional control, impulsivity, and aggression predicted BPD
Barnow et al. (42)	Longitudinal study; Community population	15 yrs 323 children	Maternal BPD as predictor of BPD onset in adolescence (15 years)
Jovev et al. (37)	Longitudinal study; Community population	11–13 yrs 245 children	Distressing childhood experiences is a significant risk factor for the development of BPD in early age.
Kaess et al. (27)	Randomized controlled study; Clinical outpatients	13–18 yrs 33 BPD 35 CC 31 HC	High harm avoidance and novelty seeking but low reward dependence represented a biological vulnerability for developing BPD
Hecht et al. (24)	Longitudinal study; Community population	11,30 yrs 314 maltreated children 285 non-maltreated children	Maltreated children were more likely to be at high risk for development of BPD
Cramer et al. (29)	Longitudinal study; Community population	11 yrs 100 children	Childhood personality traits predicted adult BPD features
Stepp et al. (33)	Longitudinal study; Community population	16–18 yrs 113 girls	Maternal BPD as predictor of BPD onset in early adulthood (around 24 years) and negative emotional reactivity predicted BPD

*BPD, borderline personality disorder; MDD, major depressive disorder; HC, healthy control; CC, clinical control; ERLTS, Environmental Risk Longitudinal Twin Study*.

## Traumatic Experiences and Early Onset of BPD

The hypothesis that early traumatic life experiences foster the development of BPD received increasing scientific validation. In particular, early traumas work as triggers for the evolution of several BPD characteristics, such as affect instability, emotion dysregulation, and self-destructive behaviors (47). Traumatic events play a central role as they seem to impair the ability of mentalizing or symbolizing emotions (48, 49), especially in early phases of life. A recent study on this focus (50) suggested that the adverse childhood experiences (ACEs) involving emotional and physical trauma, parental mental illness, and exposure to poverty in early stages of life were the strongest predictor of BPD symptoms in 14–19-year-old subjects, even when parental psychopathology and poverty were excluded from the analysis. In particular, this study found that ACEs in preschool age were particularly impacting on early development of borderline personality features.

In accordance with diathesis–stress theories of borderline etiology (13), precocious traumatic experiences in terms of abuse and neglect and inherited vulnerability (specific temperamental traits and genetic polymorphisms) play a synergistic role to foster borderline personality features. In this perspective, we cannot consider single predisposing factors to early BPD as point insults that produce the disorder by themselves, but rather as overlapping factors that added to other environmental or biological conditions contribute to the genesis of the disorder. Moreover, there is a cumulative effects of traumatic experiences: children who had experienced more than one type of abuse and maltreatment perpetrated across developmental periods had significantly higher severity of borderline personality features (24, 51–53).

We will discuss below available evidences on this issue, conceiving trauma in terms of abuse or neglect conducts from parents and peers that contributed to early development of borderline pathology. Verbal, emotional, physical, and sexual abuse, together with emotional and physical neglect, and chronic exposure to peer victimization were identified as potential factors that increase the risk for early BPD.

Some studies that will be reported in the following paragraphs are the same described in the previous section (temperamental factors and environmental factors) as authors investigated the role and interaction of different risk factors in early BPD onset.

### Sexual Abuse

Sexual abuse is defined as “any sexual act to which the victim did not consent, could not consent, or was pressured or manipulated into consenting” (54).

Most of studies showed a significant association between childhood sexual abuse (CSA) and onset of BPD at very young age (33, 50, 55–61). Nevertheless, this evidence was not replicated in all studies. In fact, Hect et al. (24) did not find a relationship between BPD and sexual abuse. This result should be interpreted considering the study limitations: a small sample size and the possibility that consequences of sexual abuse may manifest after the middle-childhood period, which was the timespan analyzed in Hect's study. Findings from several investigations suggested that adolescents with BPD have a history of sexual trauma more frequently than both healthy adolescents (57) and non-BPD psychiatric patients (58).

In BPD adolescents some authors found a higher rate of sexual abuse-related events, than in depressed and healthy control adolescents (55). In addition, sexual abuse events were significantly associated to suicidal conducts in youths with BPD (62). Psychological burden of CSA experiences does not decrease with the disclosure. On the contrary, this can be a delicate period with increased risk of suicide and development of personality disorders (60). In particular, a cohort study that compared the presence of several psychiatric diagnoses in adolescents who had been victims of CSA, reported that BPD was the only diagnosis not present at all before the disclosure of CSA experience, but it was found in the first year, and its rate drastically increased in the second year after the first registered CSA experience (61). These findings supported the specificity of the relationship between precocious sexual abuse experiences and early BPD onset.

Sexual abuse was found the only one, among different types of abuses (e.g. neglect, physical, and sexual abuse), to reach an independent significant relation with early BPD development in a study performed in a sample of adolescent females with BPD, compared with a clinical control group with mixed psychiatric diagnoses (59). Precocious sexual abuse could alter clinical manifestations and worsen the course of BPD. Some authors observed a significant relationship between sexual abuse and psychotic-like experiences in young patients with borderline personality features (63) and a more severe general clinical picture in BPD patients with prolonged childhood sexual abuse than subjects who had not experienced these traumas (50, 64). Moreover, adolescent BPD females who did not remit four years after initial presentation of symptoms were significantly more likely to have self-reported childhood sexual abuse, concomitant depressive symptoms, or substance use disorder (56). On the other hand, BPD symptoms significantly improved from ages 16 to 18 among BPD adolescent girls who did not report experiences of sexual abuses (33).

### Physical and Verbal Abuse

Physical abuse consists of “the non-accidental infliction of physical injury on the other person (e.g., bruises, welts, burns, choking, and broken bones), while verbal abuse involves some sort of verbal interaction that causes a person's emotional harm (e.g. blaming, criticism, judging, and name-calling)” (24). Several studies found a relationship between physical or verbal abuse and BPD development (23, 24, 37, 59, 65).

Children who were physically maltreated developed more BPD symptoms at age 12 compared to non-maltreated peers and were especially vulnerable if they had relatives with psychiatric disorders (23). Harsh treatment and inherited vulnerability seem to play a synergistic role to foster borderline personality features. Not only family history, but also children temperamental characteristics can facilitate manifestation of BPD symptoms if physical abuse is suffered: children with a low level of the temperamental trait of affiliation who were physically abused showed an earlier onset and a higher severity of BPD symptoms (37). The relationship between perpetrated maltreatments and temperament is complex and debated: maltreatment could promote the onset of BPD in patients who had a biological vulnerability (specific temperamental traits). Nevertheless, it is also possible that precocious and repeated familial maltreatments influence at least some of the temperamental features related to BPD. Timing of measurement of temperamental features constitutes a limitation as it is hard to distinguish temperamental traits in personality of adult patients.

The effect of physical trauma affects many domains of personality, such as affective dysregulation, identity diffusion, disturbed relationships, and self-harm. Physically abused children presented higher scores on each domain in comparison with a non-maltreated children control group. Moreover, they had higher overall borderline trait scores and were more likely to be recognized as individuals at high risk for BPD (24).

Not all studies are consistent about the specific relationship between physical abuse and early BPD development. Infurna et al. (59) found a significant association between physical abuse from father and BPD onset when univariate regression was used, while this association was lost when results were analyzed by means of a multivariate regression model, including other types of abuse experiences. Biskin et al. (56) found no difference between BPD and clinical comparison groups on self-reports of physical abuse.

As regards the effects of verbal abuse, data are limited. Some authors suggested that the impact of harsh speech can be traumatic as well as physical abuse. Johnson et al. (65) found that children who experienced verbal abuse from mother were more prone to borderline, narcissistic, obsessive-compulsive, and paranoid PDs in adolescence or early adulthood compared to subjects who did not experience verbal abuse.

### Neglect

In the context of caregiving, neglect is a kind of abuse characterized by “failure to supervise the child properly, leading to physical or emotional harm” (65). Neglect concept includes physical neglect, which is the failure to meet adequately the physical needs of children, and emotional neglect, which is represented by emotional detachment by the caregivers to the requests for attention and care of children (66).

The association between neglect and early BPD development was reported by several studies (24, 37, 57, 59, 67, 68). The most significant findings showed that: adolescents with BPD and concomitant depression had significantly higher rates of neglect than healthy controls (57); physical neglect was associated with BPD features onset at an earlier age (24); the combination between specific temperamental features and physical/emotional neglect may speed up the onset of BPD and antisocial personality disorder (ASPD) symptoms (37); and neglect from both parental figures was reported more often among BPD adolescents in comparison with other clinical populations (59).

The impact of specific kinds of neglect (i.e. childhood supervision neglect and maternal withdrawal) was investigated in two studies (67, 68). Childhood supervision neglect, including the failure to set limits, to attend to misbehavior, to know child's whereabouts and friends, was associated with higher risk for Cluster B personality disorders in adolescence and early adulthood (67). Maternal withdrawal in infancy, a kind of neglect in which mother creates physical and verbal distance from her baby, was a robust predictor of both BPD symptoms and self-injuries or suicidality in adolescence (68).

### Bullying

“Bullying is a systematic abuse of power and is defined as aggressive behavior or intentional harm doing by peers that is carried out repeatedly and involves an imbalance of power, either actual or perceived, between the victim and the bully” (69).

Bullying includes both physical and verbal acts of aggression or indirect bully victimization denoted by social exclusion (70, 71). In a psychological perspective, victimization may affect upon inner working models that pertain the relationships, disturbing the capacity to trust and interact with others in a correct manner, and leading to unstable relationships, distort perceptions, and emotional dysregulation (72).

Several studies reported that being bullied during the period of primary school is a strong predictor of early BPD onset (73, 74) approximately up to 2–6 years after the bullying conducts (73). Feelings of loneliness, anger, and loss of trust that are due to victimization by peers were frequently described by victims of bullying who had developed BPD in young age and were observed also during experimental social trust games (75, 76).

Wolke and colleagues (73) conducted one study in order to explore the association between peer victimization and BPD symptoms. Authors considered peer victimization during primary school as a potential predictor of the disorder's onset at about 12 years and observed that children who were exposed to chronic bullying (at 8 and 10 years) had an augmented risk of BPD symptoms with a dose-response relationship. Children who experienced relational and repeated peer victimization had seven times increased risk to early BPD symptoms compared to those that were not bullied. In our previous investigation aimed to identify which factors are independently associated to early onset of BPD, we found that earlier onset of BPD is associated to traumatic experiences, including abuse, neglect, and dysfunction in household environment. A significant traumatic condition that was identified in our analysis of early BPD risk factors is bully victimization (74). Studies on precocious traumas (sexual abuse, physical/verbal abuse, neglect, bullying) and early onset BPD are displayed in Table 2.

**Table 2:** Studies on precocious traumas (sexual abuse, physical/verbal abuse, neglect, and bullying) and early onset BPD.

**Sexual abuse**	**Study design**	**Recruitment age (mean or range) /patients (*n*)**	**Outcomes**
Horesh et al. (55)	Controlled trial; Clinical outpatients and community population	16,26 yrs old (mean) 19 MDD 20 BPD 20 HC	Sexual abuse more frequent in BPD than MDD and HC
Horesh et al. (62)	Retrospective study; Clinical outpatients and community population	13–21 yrs old 22 MDD suicidal 18 BPD suicidal 20 MDD non- suicidal 20 BPD non suicidal 40 HC	Sexual abuse associated with suicidal BPD
Goodman et al. (57)	Controlled trial; Clinical outpatients and community population	16 yrs old (mean) 13 HC 13 MDD/BPD	Sexual abuse associated with MDD/BPD patients
Biskin et al. (56)	Longitudinal study; clinical outpatients and community population	19,6 yrs old (mean) 31 BPD 16 HC	BPD girls without remission after 4 years from presentation more likely report CSA, current MDD or substance use disorder
Venta et al. (58)	Controlled trial; Clinical inpatients	16,04 yrs old (mean) 34 BPD 113 CC	Sexual abuse more likely in BPD than CC
Infurna et al. (59)	Retrospective study; clinical outpatients	15,57 yrs old (mean) 44 BPD 47 CC	Sexual abuse predicted BPD development
Stepp et al. (33)	Longitudinal study; Community population	16 yrs old (mean) 113 adolescent girls	BPD symptoms significantly decreased from ages 16–18 among BPD adolescent girls without CSA history.
Kaplan et al. (60)	Longitudinal study; Clinical outpatients	13–21 yrs old 29 abused BPD 29 not-abused BPD	Sexual abuse associated with suicidal BPD
Sengutta et al. (63)	Retrospective study; Clinical inpatients	16–21 yrs old 200 non-psychotic patients	Strong link between sexual abuse and psychotic-like experiences in young patients with BPD features
Turniansky et al. (64)	Retrospective study; Clinical outpatients	11–19 yrs old 38 BPD sexual abused 40 BPD without sexual abuse	BPD patients with a history of prolonged CSA manifest more severe clinical presentation compared to those without prolonged CSA
Geselowitz et al. (50)	Longitudinal study; Clinical outpatients and community population	14–19 yrs old 41 BPD 129 HC	Pre-school ACEs predicted BPD symptoms.
Rajan et al. (61)	Longitudinal study; Community population	12–17 yrs old 519 abused girls 4,920 HC	Strong link between BPD and sexual abuse
**Physical/verbal abuse**	**Study design**	**Recruitment age/patients(** ***n*** **)**	**Outcomes**
Johnson et al. (65)	Longitudinal study; Community population	5,5 yrs old 793 children	Strong relation between verbal abuse and BPD development
Biskin et al. (56)	Longitudinal study; Clinical outpatients and community population	19,6 yrs old (mean) 31 BPD 16 HC	No link between physical abuse and BPD symptoms
Belsky et al. (23)	Longitudinal study; Community population (ERLTS)	At birth 2,232 twins	Physical abuse predicted BPD symptoms
Jovev et al. (37)	Longitudinal study; Community population	11–13 yrs old 245 children	Abuse associated with BPD symptoms for children with low affiliation
Hecht et al. (24)	Retrospective study; Community population	10–12 yrs old 11,3 yrs old (mean) 314 maltreated children 285 non-maltreated children	Physical abuse predicted BPD symptoms
Infurna et al. (59)	Retrospective study; Clinical outpatients	15,57 yrs old (mean) 44 BPD 47 CC	Physical abuse from father predicted BPD development (univariate regression model only)
**Neglect**	**Study design**	**Recruitment age/patients(** ***n*** **)**	**Outcomes**
Johnson et al. (67)	Longitudinal study; Community population	1–10 yrs old 738 children	Supervision neglect predicted BPD symptoms
Goodman et al. (57)	Controlled trial; Clinical outpatients and community population	16 yrs old (mean) 13 HC 13 MDD/BPD	Neglect associated with MDD/BPD patients
Lyons-Ruth et al. (68)	Longitudinalstudy; Community population	1,5 yrs old 56 children	Maternal withdrawal in infancy predicted BPD symptoms and suicidality/self-injury in late adolescents
Jovev et al. (37)	Longitudinal study; Community population	11–13 yrs old 245 children	Neglect predicted BPD symptoms
Hecht et al. (24)	Retrospective study; Community population	10–12 yrs old 11,3 yrs old (mean) 314 maltreated children 285 non-maltreated children	Physical neglect predicted BPD symptoms
Infurna et al. (59)	Retrospective study; Clinical outpatients	15,57 yrs old (mean) 44 BPD 47 CC	Physical abuse from father predicted BPD development (univariate regression model only)
**Bullying**	**Study design**	**Recruitment age/patients (** ***n*** **)**	**Outcomes**
King-Casas et al. (76)	Controlled trial; Clinical outpatients and community population	55 BPD 38 HC	BPD patients have difficulty to trust others
Wolke et al. (73)	Longitudinal study; Community population (ALSPAC)	4-10 yrs old 6050 children	Abuse by adults and bullying predicted BPD
Takizawa et al. (77)	Longitudinal study; Community population	7-11 yrs old 7771 children	Bullying predicted MDD, anxiety disorders and suicidality

*BPD, borderline personality disorder; MDD, major depressive disorder; CSA, childhood sexual abuse; HC, healthy control; CC, clinical control; ACE, adverse childhood experience; ERLTS, Environmental Risk Longitudinal Twin Study; ALSPAC, Avon Longitudinal Study of Parents and Children*.

## Neurobiology, Neuro-Morphology and Epigenetic in Early Trauma and BPD

There is a general consensus in retaining that alterations in several biological systems and brain anatomical features are related to traumas in childhood and to early BPD. Some authors have examined the effect of early stressful events on biological systems in order to identify biomarkers that could be involved in BPD vulnerability. These markers might allow an early detection of individuals at high risk for BPD (2).

Precocious traumatic experiences lead to chronic “hyperarousal” of the hypothalamic-pituitary- adrenal axis (HPA), resulting in higher levels of cortisol. Several investigators found an increase of cortisol levels (urinary, salivary, and blood) in patients with BPD, hallmarking HPA activation (78–80). The cortisol hyper-stimulation of the hippocampus can generate a distorted interpretation of signals from the environment as constant menaces, therefore sending this danger signals to amygdala, that modulates fear and aggression (20). Intense emotional responses to small stress with a greater latency for returning to baseline conditions are common in young BPD patients. Moreover, over-functioning of the prefrontal cortex generates loss of rationality, reasoning, and decision- making capacity in BPD patients (20, 81).

With regards to neuroimaging investigations, several preclinical studies have delineated the effects of early stressors on specific brain regions. Corpus callosum and other myelinate areas are potentially vulnerable to the impact of early exposure to higher levels of stress hormones, that suppress glial cell division crucial for myelination (82) and determine a reduced size of the corpus callosum (83, 84). These results were confirmed in clinical investigations. De Bellis and colleagues observed the reduction of volume of corpus callosum size in children with a history of abuse and post-traumatic stress disorder (PTSD) (85). Reduction of the corpus callosum size may lead to a diminished communication between the hemispheres, with an increased hemispheric laterality (82).

The association between decreased hippocampal volume, early traumatic events, and development of psychiatric disorders in early phases of life was also investigated (86, 87). In particular, an average of 16% reduction in hippocampal volume bilaterally was seen in BPD females with a history of childhood victimization (88). Some authors observed a direct proportional correlation between the amount of volume reduction in the mentioned areas and severity of trauma exposure in childhood (89). In addition to the volumetric reduction that was observed in corpus callosum, hippocampus, and prefrontal cortex, some abnormalities in activity were found in amygdala, in particular in BPD females with traumatic experiences in childhood (90).

Despite the detrimental consequences of child maltreatment on developmental processes, some subjects show resilience, with low level of psychopathology, while others develop severe disorders. Gene-environment interaction model can help us to better understand the role of trauma on genetic predisposition to develop early BPD. An interconnection between genes and environment in relation to behavior was firstly reported by Caspi et al. (91). In particular, authors observed that maltreated children who developed conduct disorder and antisocial personality disorder had a genotype that resulted in low levels of monoamine oxidase A (MAOA) expression (92).

Some studies evaluated how serious life events (SLE) (i.e., physical abuse, rape, and childhood sexual abuse—CSA) interact with polymorphisms of genes in producing behavioral patterns characteristic of BPD. In BPD patients trauma was found associated with a decrease or an increase of impulsivity depending on the genotype: lower impulsivity after SLE was associated to SS and SL genotypes and higher impulsivity was associated to LL carriers of the long/short (L/S) polymorphism of the serotonin transporter promoter (5-HTTLPR) (93). Interactions between trauma and the rs4680 COMT polymorphism involved in dopaminergic functioning (COMT Val158Met) was also evaluated. Results reported that the Val/Val genotype (but not the Val/Met and Met/Met genotypes) was associated with lower impulsive aggression in BPD (94).

Martin Blanco and colleagues (95) performed a study aimed to study the interaction of HPA genetic polymorphisms and childhood trauma in BPD. Authors found that two FKBP Prolyl Isomerase 5 (FKBP5) alleles (rs3798347-T and rs10947563-A) were more frequent in BPD subjects who experienced physical abuse and emotional neglect and two Corticotropin-releasing factor receptor 2 (CRHR2) variants (rs4722999-C and rs12701020-C) were more common in BPD subjects sexually and physically abused (95). These findings can suggest that childhood trauma modulates the onset of this disorder (92).

In order to better understand the influence of the environment on gene, some epigenetic studies were performed. Perroud et al. (96) investigated epigenetic changes of serotonin 3A receptor (5-HT3AR) in patients with childhood maltreatment and several psychiatric disorders. They found that epigenetic modification of 5-HT3AR was related to a history of childhood maltreatment and more severe psychiatric disorders, including BPD, in adulthood (96). Another study showed a higher methylation of glucocorticoid receptor gene NR3C1 in patients with BPD and childhood traumas (97).

Studies on neurobiological, neuro-morphological, and epigenetic factors in early onset BPD are described in Table 3.

**Table 3:** Studies on neurobiological, neuro-morphological, and epigenetic factors in early onset BPD.

**Neurobiology, neuro- morphology and epigenetic factors**	**Study design**	**Recruitment age (mean or range)/patients (*n*)**	**Outcomes**
de Bellis et al. (85)	Case-control study; MRI brain scan; Clinical outpatients and community population	44 maltreated children PTSD 61 HC	Reduced corpus callosum size in children with a history of abuse and PTSD
Driessen et al. (88)	Case-control study; MRI brain scan; Clinical outpatients and community population	20–35 yrs 21 BPD victimized 21 HC	BPD patients with a history of victimization had an average 16% reduction in hippocampal volume
Herpertz et al. (90)	Case-control study; fMRI brain scan; Clinical outpatients and community population	26.2 yrs (mean) 6 BPD 6 HC	Increased blood oxygenation levels of amygdala and activation of the medial and inferolateral prefrontal cortex in BPD with childhood trauma
Rinne et al. (79)	Dexamethasone/CRH (DEX/CRH) test; Clinical outpatients and community population	18–50 yrs 39 BPD 11 HC	Increased urinary, salivary, or blood cortisol levels in individuals with BPD
Lieb et al. (78)	Case-control study; dexamethasone suppression test (DEX); Clinical outpatients and community population	19–47 yrs BPD HC	Increased urinary, salivary and blood cortisol levels in individuals with BPD
Wagner et al. (93)	Case-control study; Clinical outpatients and community population	33 yrs 161 BPD 161 HC	BPD patients trauma associated with a decrease or an increase in impulsivity depending on the genotype (5-HTTLPR; COMT Val158Met)
Kuhlmann et al. (97)	Case-control study; MRI brain scan; Clinical and outpatients community population	18–35 yrs 30 BPD 33 HC	Higher methylation of NR3C1 in patients with BPD with childhood trauma
Martín-Blanco et al. (95)	Case-control study; Clinical outpatients and community population	30,05 yrs (mean) 481 BPD 442 HC	FKBP5 alleles (rs3798347-T and rs10947563-A) and CRHR2 variants (rs4722999-C and rs12701020-C) were more common in BPD with sexual and physical abuse
Perroud et al. (96)	Case-control study; Clinical outpatients and community population	31,5 yrs (mean) 116 BPD 111 ADHD 122 BD	Epigenetic modification of 5- HT3AR linked to history of childhood maltreatment in BPD

*BPD, borderline personality disorder; MDD, major depressive disorder; HC, healthy control; CC, clinical control; PTSD, post-traumatic stress disorder; SLE, serious life events; MRI, magnetic resonance imaging; fMRI, functional magnetic resonance imaging; BP, bipolar; NR3C1, Nuclear Receptor Subfamily 3, Group C, Member 1 (Glucocorticoid Receptor); FKBP5, FK506 binding protein 5; CRHR2, Corticotropin Releasing Hormone Receptor 2; 5-HT3AR, **5**-Hydroxytryptamine Receptor 3A; 5- HTTLPR, serotonin-transporter-linked promoter region; COMT, Catechol-O-methyltransferase*.

## BPD and Complex PTSD: Recent Traumatic Experiences Could Reactivate Precocious Traumas?

Repeated traumatic events provoked by caregivers or intimate partners involve the risk of developing a constellation of psychiatric symptoms. Complex Post Traumatic Stress Disorder (cPTSD) was originally conceptualized to indicate the reaction to multiple, perpetrated traumatic events with onset in early life (98). More recently the diagnosis of cPTSD referred to a “clinical syndrome following experiences of interpersonal traumatic victimization and is characterized by difficulties in emotion regulation, interpersonal relationships, and self-concept” (99). So, cPTSD shows some overlapping with BPD, including dissociative symptoms, dysregulation of emotions, self and relational disturbances (100). In fact, chronic precocious traumatic experiences often result in more pervasive disorders than simple PTSD with impairment in attachment style and in the ability to modulate emotions. Trauma can produce its effects on behavioral, emotional, physiologic, and neuroanatomical levels. Assaults lead to hyperarousal states that can interfere with right judgment of the relationships and situations. Later stressors tend to be experienced by victims of trauma as a reactivation of the precocious traumatic experiences (101).

Prolonged and repeated traumas, particularly in early life, promote a chronic inability to modulate emotions, that can result in behavioral patterns characteristic of BPD, such as disturbed relationships, substance abuse, and self-injuries behaviors, in which precocious traumatic events are re-enacted over time (102).

Despite the great interest in this topic, systematic investigations focused on the relationship between precocious traumas, present traumatic experiences, complex post traumatic disorder and BPD psychopathology are still scarce. One of the main reasons is that complex PTSD has been proposed by several investigators, but has been included as an official diagnosis only very recently in ICD-11. So, only now well-defined diagnostic criteria are available for PTSD and BPD in DSM- 5 and for cPTSD in ICD-11. A comparison of these criteria in clinical samples will allow to identify more clearly differences and similarities of these psychopathological constructs.

One study was performed in order to determine whether patterns of affective dysregulation, relational impairment, and identity alterations, mediate the relationship between exposure to interpersonal traumatic stressors in childhood and present clinical symptoms in a sample of adults diagnosed with severe cPTSD/BPD. Results of the study demonstrated that the relationship between childhood exposure to trauma and cPTSD/BPD symptoms in adulthood was mediated by the three factors: relational impairment, affect dysregulation, and identity alterations (103). Some authors hypothesized that emotion dysregulation and impulsivity in BPD may increase vulnerability to be re-exposed to traumatic events due to high appraisal of threat, diminished coping resources, increased exposure to risky situations, and intense emotional responding peri-traumatically (104, 105). Exposure to multiple traumatization affected the sense of self and emotion regulation strategies (106). These key alterations of personality can evolve into BPD or cPTSD. Emotional dysregulation may also increase the tendency to perceive new events as threatening and traumatic (107).

Studies on BPD and complex PTSD are presented in Table 4.

**Table 4:** Studies on BPD and complex PTSD.

**BPD and CPTSD**	**Study design**	**Recruitment age (mean or range)/patients (*n*)**	**Outcomes**
Orbach et al. (106)	Longitudinal study; clinical inpatients	25–60 yrs 32.43 yrs (mean) 32 suicidal BPD 29 non-suicidal BPD	Exposure to multiple traumatization affected the sense of self and emotion regulation strategies
Bardeen et al. (105)	Longitudinal study; Community population	18 yrs 145 girls	Emotion dysregulation and impulsivity in BPD re-exposed to traumatic events
van Dijke et al. (103)	Retrospective study; Clinical outpatients	34.7 years 472 PD	Relationship between childhood trauma and cPTSD/BPD mediated by elational impairment, affect dysregulation, and identity alterations

*BPD, borderline personality disorder; cPTSD, Complex Post Traumatic Stress Disorder; HC, Healthy control; CC, Clinical control; PD, personality disorder*.

## Conclusions

On the basis of the results discussed in the previous sections, we can conclude that the interaction of temperamental, environmental, and genetic factors with early traumatic experiences can promote onset of BPD in young age. Available data suggested that experiences of abuse, neglect, and bully victimization in childhood, temperamental traits of impulsive aggression and negative affectivity interacting with dysfunctional familial environment, volumetric and functional abnormalities in fronto-limbic areas associated to precocious trauma and specific polymorphisms of genes (MAOA; 5-HTTLPR; FKBP5; CRHR2; 5HTTR; COMT) characterize subjects at high risk to develop BPD. In accordance with our preliminary model of risk factors in early BPD (3), the effect of the interaction of different risk factors is more decisive than the separate effects of single factors in early development of BPD. So, the effects of traumatic experiences are enhanced when the dysfunctional familial environment that produces traumas interacts with the child's innate temperamental traits or specific genetic polymorphisms.

The mechanisms of interaction of different predisposing factors are only partially known and further studies are required to understand which factors have an independent effect and which produce their action as mediators or modulators.

## Author Contributions

All authors provided their contribution to collection and analysis of data and preparation of manuscript.

## Conflict of Interest

The authors declare that the research was conducted in the absence of any commercial or financial relationships that could be construed as a potential conflict of interest.

## Publisher's Note

All claims expressed in this article are solely those of the authors and do not necessarily represent those of their affiliated organizations, or those of the publisher, the editors and the reviewers. Any product that may be evaluated in this article, or claim that may be made by its manufacturer, is not guaranteed or endorsed by the publisher.
